# Intramedullary Nail Failure in a Subtrochanteric Fracture in a 62-Year-Old Woman

**DOI:** 10.7759/cureus.75485

**Published:** 2024-12-10

**Authors:** Benjamin T Lack, Justin T Childers, Charles H Hennekens, Jonathan B Courtney

**Affiliations:** 1 Department of Surgery, Florida Atlantic University Charles E. Schmidt College of Medicine, Boca Raton, USA; 2 Department of Medicine, Florida Atlantic University Charles E. Schmidt College of Medicine, Boca Raton, USA; 3 Department of Population Health and Social Medicine, Florida Atlantic University Charles E. Schmidt College of Medicine, Boca Raton, USA

**Keywords:** hip fracture, implant failure, intramedullary nail, orthopedic trauma, subtrochanteric fracture

## Abstract

Subtrochanteric fractures in older patients are typically due to low-energy falls. The standard of care is intramedullary nailing. The Smith & Nephew Trigen Intertan (Memphis, TN, US) is an intramedullary nail with a novel design that incorporates two integrated compression screws. We present a case of intramedullary nail failure in a patient with a subtrochanteric fracture. A 62-year-old woman with osteoporosis, a 40-year history of smoking, and consumption of two to three alcoholic beverages daily, weighing 177 pounds and five feet two inches tall (BMI 32.4), experienced a low-energy fall in her home. She presented to the emergency department (ED) with a shortened and externally rotated right leg with pain on manipulation. Her neurovasculature was intact. An X-ray revealed a comminuted, right subtrochanteric femur fracture. The patient underwent open reduction and internal fixation with a Trigen Intertan nail with no intraoperative complications. Her leg returned to pre-injury length, and she resumed ambulating at her pre-injury level. Six weeks later, the patient experienced pain upon standing. She was transported to the ED by ambulance where she presented with a shortened externally rotated right leg with pain on manipulation. An X-ray revealed failure of the intramedullary nail with breakage at the lag screw hole. Her care was transferred to another orthopedic surgeon (JC), where she underwent hardware removal and conversion to a total hip arthroplasty. Postoperatively, her legs were of equal length, and her neurovasculature remained intact. Four months later, at her final follow-up, she was ambulating unassisted at her pre-fracture level. Subtrochanteric fractures in elderly patients pose serious threats to morbidity, mortality, and quality of life. Surgery with intramedullary nail placement and rapid ambulation have generally favorable outcomes. Failure rates are generally very low. Such circumstances generally require hardware removal and total hip arthroplasty, which are generally curative but can confer small risks of morbidity and mortality.

## Introduction

Subtrochanteric fracture of the femur represents between 7% and 34% of femur fractures [1]. These injuries have a bimodal incidence, traditionally presenting in younger patients due to high-energy traumas such as motor vehicle collisions and in elderly patients due to low-energy falls [1,2]. Fractures of this nature can be devastating to the quality of life in elderly patients. The current standard of care for repairing a subtrochanteric fracture is intramedullary fixation [3]. The type of intramedullary nail that is used in fixation is an important surgical consideration as nails vary in length, size, and mechanism. The consequences of the type of nail selected can impact the future likelihood of failure of the implant. One case series found that the aggregate failure rate of the most frequently used intramedullary nails currently available is 0.4% [4]. The Smith & Nephew Trigen Intertan (IT) (Memphis, TN, US) is an intramedullary nail with a somewhat novel design, incorporating two integrated compression screws [5]. This active compression helps to resist strain at the fracture line to prevent fracture malunion. The compression screw markedly reduces the possibility of medial migration. This effectively minimizes the possibility of the Z effect. This effect results from the migration of screws in opposite directions, which may lead to the migration of screws in opposite directions and subsequent failure of osteosynthesis. This design, while potentially advantageous, in theory, may be more difficult to implement in practice. One review compared the outcomes of IT with two popular single-nail systems, the Proximal Femoral Nail Antirotation (PFNA) system (Synthes, Oberdorf, Switzerland) and Gamma 3 (Stryker, Kalamazoo, MI, US) [5]. Specifically, IT was associated with more favorable outcomes compared to single-screw designs including a lower incidence of secondary periprosthetic fracture, screw loosening and migration, and lower rates of the collapse of the neck-shaft angle into the varus position leading to extrusion of screws from the femoral head. This phenomenon is sometimes referred to as cut-out. To the best of our knowledge, data is sparse about the rare occurrence of screw failure requiring revision surgery following an IT implant. We present a case of a 62-year-old woman with a right subtrochanteric fracture who underwent intramedullary nail placement with an IT implant that failed after six weeks and required hardware removal and arthroplasty.

## Case presentation

**Figure 1:**
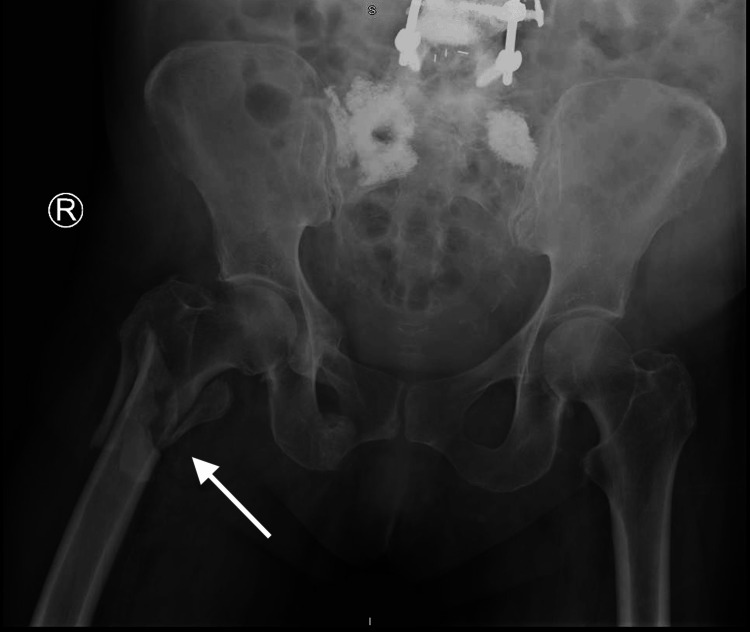
AP Pelvis Injury White arrow: right comminuted subtrochanteric femur fracture

**Figure 2:**
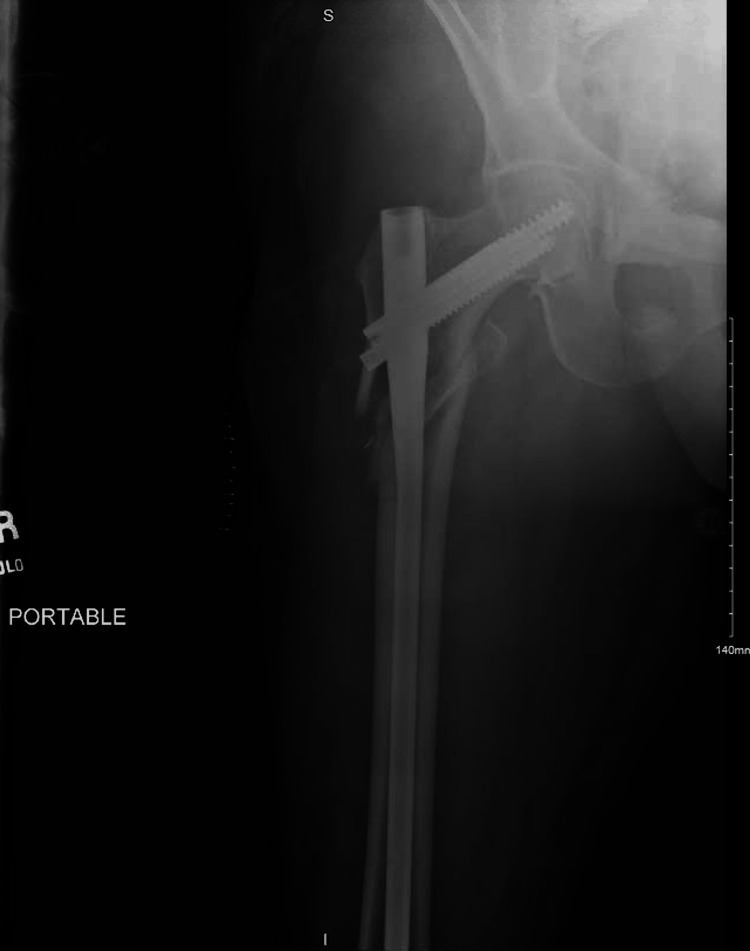
AP Femur Postoperative

A 62-year-old woman with osteoporosis, a 40-pack/year history of cigarette smoking, and consumption of two to three alcoholic beverages daily for more than 10 years, weighing 177 pounds and five feet two inches tall (BMI of 32.4), fell in her home. She required an ambulance to take her to the emergency department (ED) where she presented with an externally rotated and shortened right leg with pain on any manipulation and an inability to bear weight. Neurovascularity was intact. After her initial examination, the patient was taken for a plain radiograph, which revealed a comminuted, right subtrochanteric femur fracture (Figure 1). She was then transported to the operating room, where she underwent closed reduction and internal fixation with an Intertan nail by the on-call orthopedic surgeon. No intraoperative complications were reported (Figure 2).

**Figure 3:**
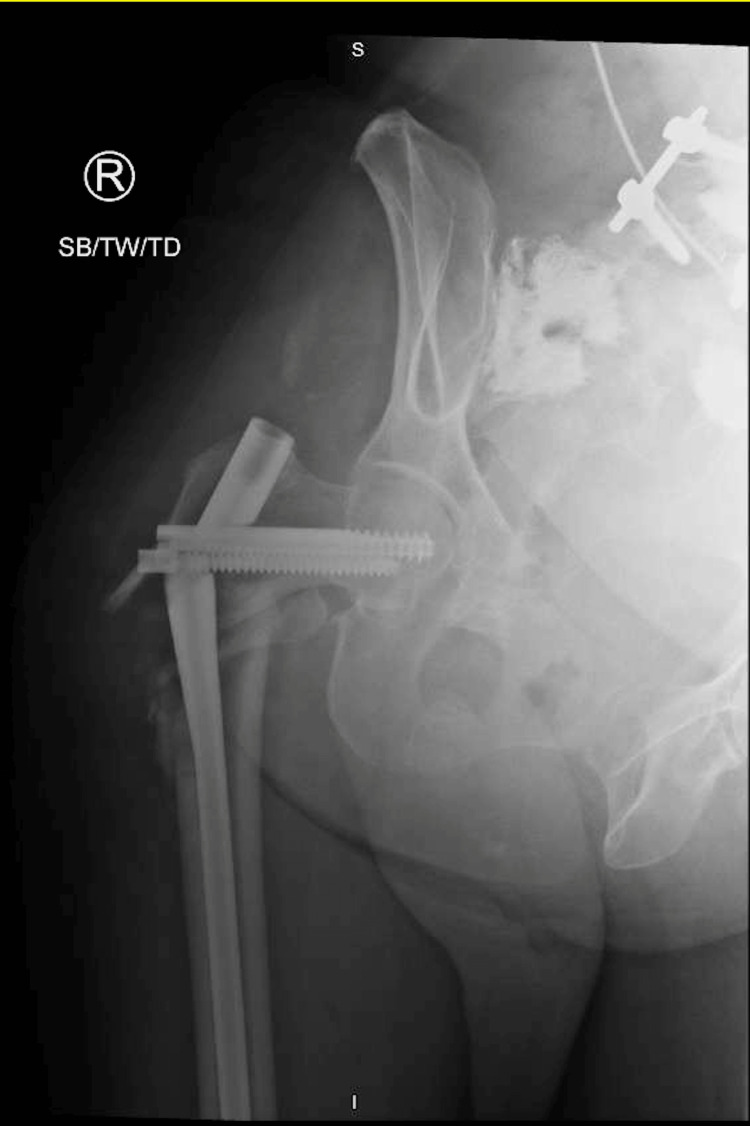
AP Femur Fracture and Nail Failure

**Figure 4:**
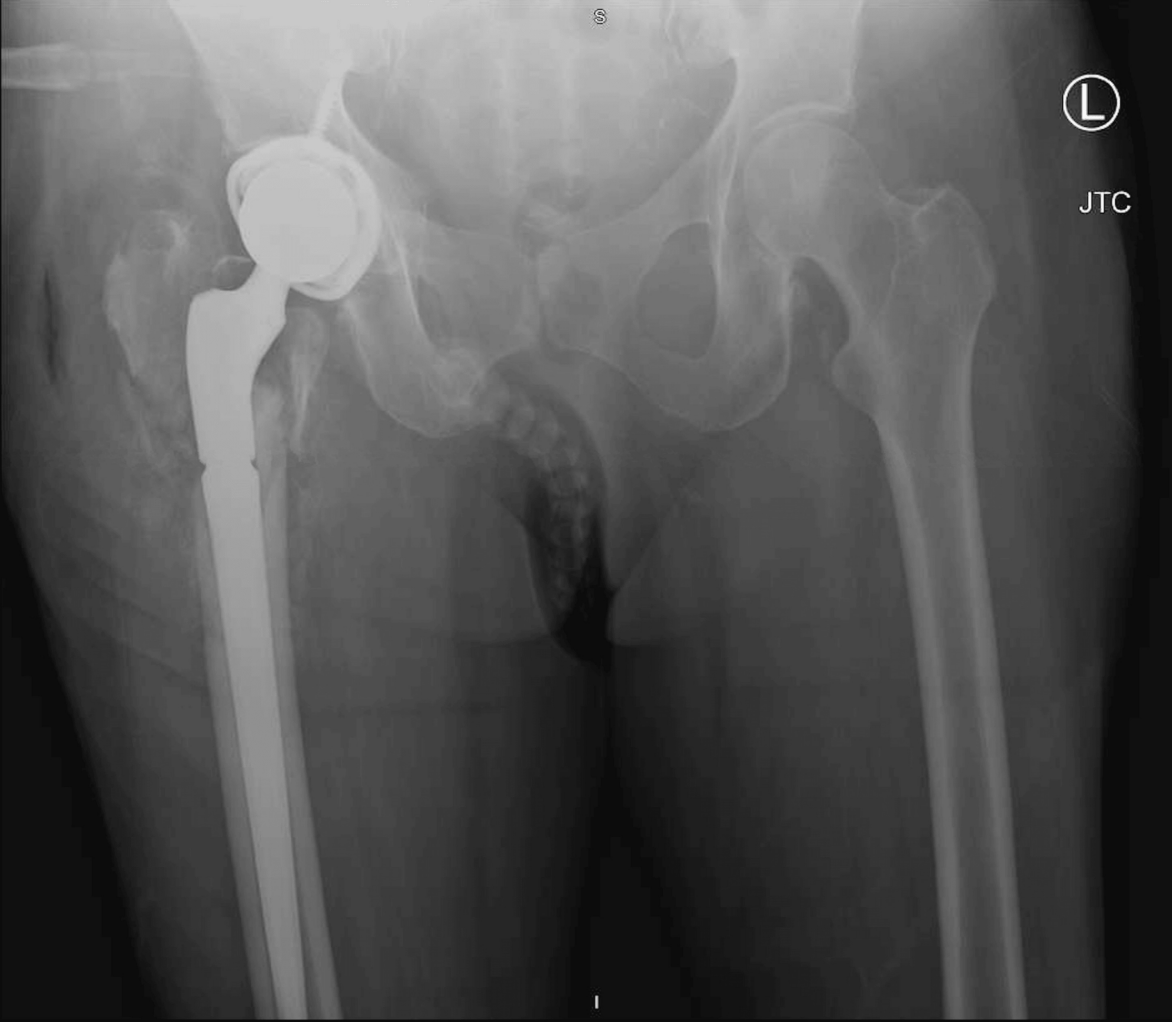
AP Pelvis Following Total Hip Arthroplasty

Following surgery, the patient began to present with symptoms of delirium tremens because of alcohol withdrawal. The patient was subsequently admitted to the hospital’s surgical intensive care unit (SICU) where she spent eight days undergoing treatment with phenobarbital for benzodiazepine-resistant alcohol withdrawal. Two weeks from the date of her surgery, she was discharged from the hospital. As assessed at the two- and six-week follow-ups, her leg returned to pre-injury length, and the incision healed as expected. She was able to resume ambulating at a pre-injury level. At that time, the patient developed pain upon standing from a seated position. She reported hearing a “pop” as she stood up, which was accompanied by intense pain in her right hip that caused her to fall back into the chair. She was transported by ambulance to the ED where she presented with severe pain on any manipulation as well as a shortened and externally rotated right leg with intact neurovascularity. An X-ray revealed failure of the intramedullary nail with breakage at the lag screw hole (Figure 3). The patient’s care was transferred to another orthopedist (JC) who performed hardware removal and a total hip arthroplasty via a posterior approach (Figure 4).

**Figure 5:**
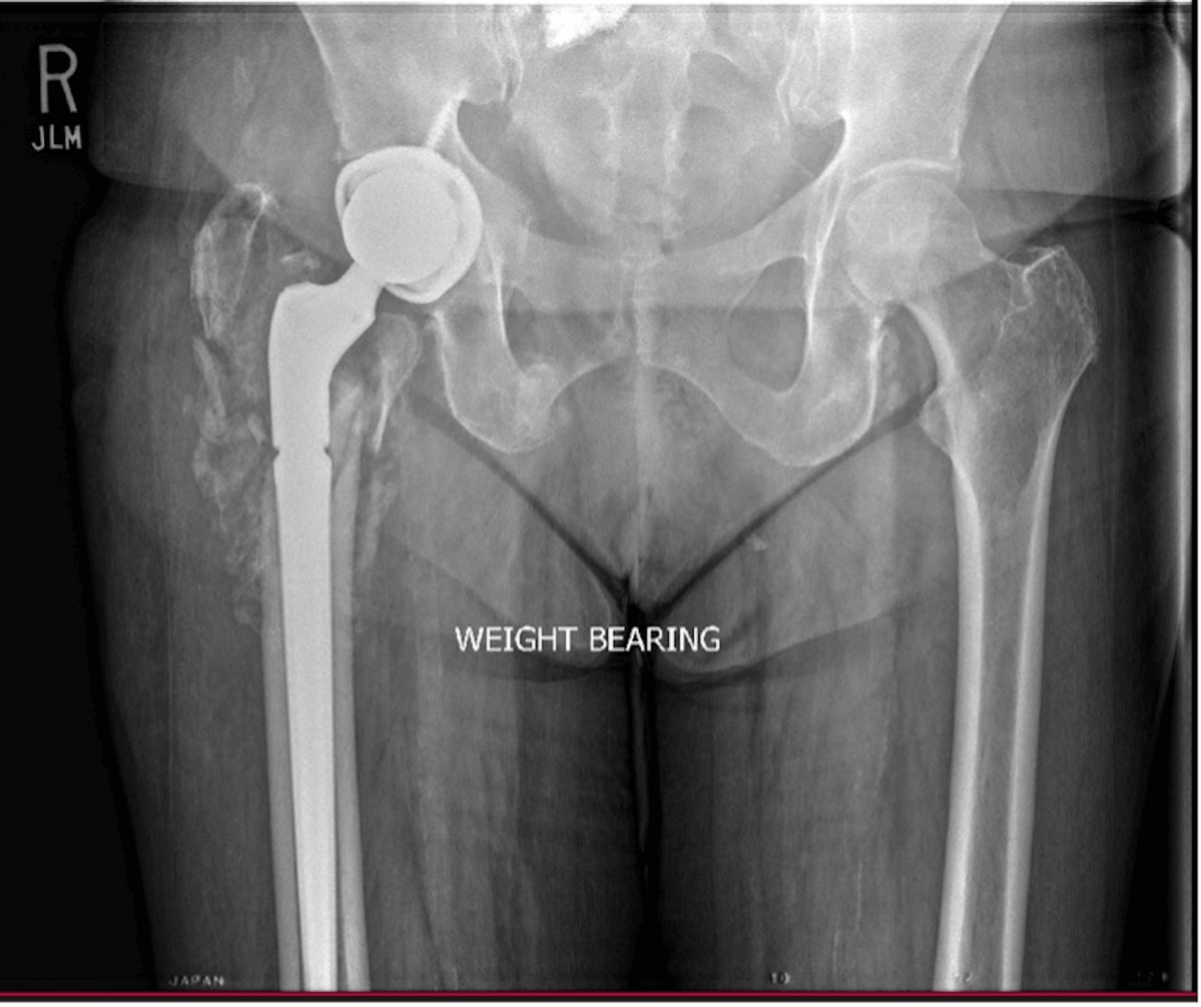
AP Pelvis Six Weeks Following Total Hip Arthroplasty

**Figure 6:**
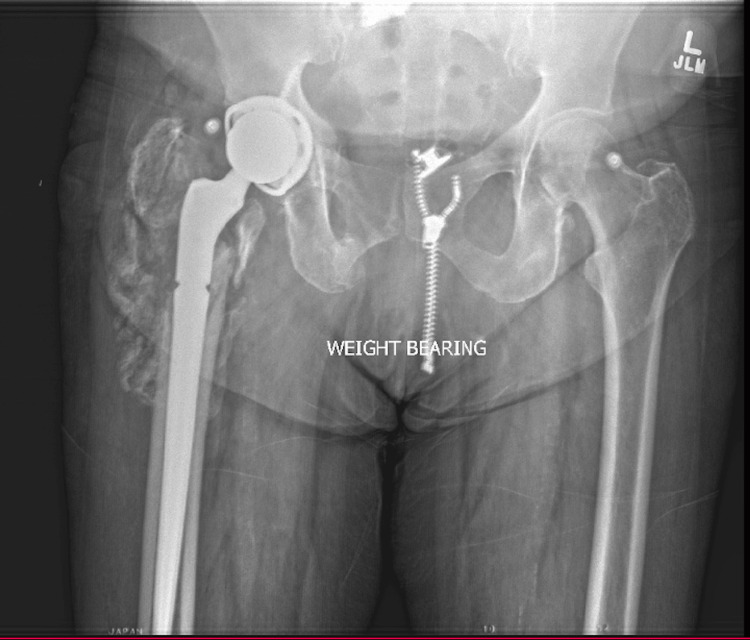
AP Pelvis Final Postoperative Visit

Postoperatively, her legs were of equal length, and her neurovascularity remained intact. At the six-week follow-up, she was ambulating without assistance (Figure 5). At her final visit, four months post-op, she was walking without assistance at her pre-fracture level (Figure 6).

## Discussion

This case is a middle-aged woman with a primary comminuted, right subtrochanteric femur fracture who underwent revision surgery and conversion to a total hip arthroplasty due to mechanical failure of the Trigen IT nail. The IT nail utilizes two cephalocervical screws, allowing for increased resistance to femoral head rotation and increased linear compression [5]. Failure of any intramedullary nail remains a rare but serious complication of open reduction with internal fixation treatment of proximal femoral fractures. In a case series of 1,481 patients undergoing intramedullary nailing for repair of femur fractures, there were 13 instances of implant failures, leading to an aggregate rate of 0.9% [4]. Of these failures, eight were reported in cases involving the usage of Intertan.

In one case, the usage of IT in the repair of an intertrochanteric femur fracture resulted in mechanical failure requiring hardware removal [6]. In this patient, breakage occurred at the lag screw hole resulting in the failure of the compression screw. Mechanical failure of this nature is a very rare but serious complication and is costly to both patient and provider. In a large multicenter study comparing IT nails with sliding hip screws in the treatment of intertrochanteric and subtrochanteric fractures, the two treatment modalities were found to have similar outcomes in pain, functionality, and mobility [7]. The time to implant breakage in the patient identified in this case was less than two months. In a previous report of 38 cases of nail failure, the mean time was about nine months with a range from three to 24 [8].

Due to its dual-screw design, the IT nail requires additional drilling and longer operating times than conventional nails [5]. It is important to assess whether the additional surgical undertaking required to perform IT nail implantation results in significant improvement in patient outcomes. Further analytic studies are necessary, which optimally would include direct randomized comparisons of all the different intramedullary nails [9,10].

## Conclusions

Subtrochanteric fractures in elderly patients pose serious threats to morbidity, mortality, and quality of life. Surgery with intramedullary nail placement and rapid ambulation have generally favorable outcomes. Failure rates are generally very low. Such circumstances generally require hardware removal and total hip arthroplasty, which are generally curative but can confer small risks of morbidity and mortality.
